# Hybrid management, organizational configuration, and medical professionalism: evidence from the establishment of a clinical directorate in Portugal

**DOI:** 10.1186/s12913-016-1398-2

**Published:** 2016-05-24

**Authors:** T. Correia, J. L. Denis

**Affiliations:** Instituto Universitário de Lisboa (ISCTE-IUL), Lisbon, 1649-026 Portugal; École Nationale d’Administration Publique, Montreal, QC H2T 3E5 Canada

**Keywords:** Hybrid management, Clinical directorate, Divisionalized professional bureaucracy, Organized professionalism, Balkanized-organized professionalism, Portugal

## Abstract

**Background:**

The need of improving the governance of healthcare services has brought health professionals into management positions. However, both the processes and outcomes of this policy change highlight differences among the European countries. This article provides in-depth evidence that neither quantitative data nor cross-country comparisons have been able to provide regarding the influence of hybrids in the functioning of hospital organizations and impact on clinicians’ autonomy and exposure to hybridization.

**Methods:**

The study was designed to witness the process of institutional change from the inside and while that process was underway. It reports a case study carried out in a public hospital in Portugal when the establishment of a clinical directorate was being negotiated. Data collection comprises semi-structured interviews with general managers and surgeons complemented with observations.

**Results:**

The clinical directorate under study illustrates a divisionalized professional bureaucracy model that combines features of professional bureaucracies and divisionalized forms. The hybrid manager is key to understand the extent to which practising clinicians are more accountable and to whom given that managerial tools of control have not been strengthened, and trust-based relations allow them to keep professional autonomy untouched. In sum, clinicians are allowed to profit from their activity and to perform autonomously from the hospital’s board of directors. The advantageous conditions enjoyed by the clinical directorate intensify internal re-stratification in medicine, thus suggesting forms of divisionalized medical professionalism grounded in organizational dynamics.

**Conclusion:**

It is discussed the extent to which policy change to the governance of health organizations regarding the relationship between medicine and management is subject to specific constraints at the workplace level, thus conditioning the expected outcomes of policy setting. The study also highlights the role of hybrid managers in determining the extent to which practising professionals are more accountable to managerial criteria. The overall conclusion is that although medical and managerial values link to each other, clinicians reconfigure managerial criteria according to specific interests. Ultimately, medical autonomy and authority may be reinforced in organizational settings subject to NPM-driven reforms.

## Background

The need of improving the governance of healthcare services has brought health professionals into management positions, thereby performing hybrid management roles [1, 2]. Professional and managerial knowledge is growingly perceived as complementary [3], and more evidence has showed that hybrid managers enhance clinical and management outcomes [4, 5]. Frequently the medical profession has supported this policy change by encouraging skill acquisition in medical leadership, therefore making doctors keen on quality, innovation and performance [6, 7]. In sum, hybrids are blurring traditional professional identities and models of work organization.

However, both the processes and outcomes of this policy change highlight differences across countries [8, 9]. One variation concerns the types of hybrids experienced in different organizational structures [10, 11]. Another variation concerns the extent to which clinicians have become accountable to external players, including patients, allied health professionals, and management itself [12–15].

Despite the significant growth of the literature on hybrid management, detailed attention is still necessary as regards micro-level processes of role configuration and relations among hybrids and their medical peers. This article goes in this direction, in particular by focusing on the process of organizational change involving hybrid management roles. Drawing on the case of clinical directorates (CDs) in Portugal, the analysis intends to provide evidence on how hybrid roles impact on practising clinicians’ autonomy and exposure to hybridization. Furthermore, few attempts have been made to incorporate CDs into the literature on organizational configuration. The relevance of the Portuguese case lies in the distinctive traits of health reforms compared to other known Anglo-Saxon and Scandinavian countries.

The article is structured as follows. First, we focus on the broad discussion of hybrid management roles in the health sector. Second, CDs are described as the finest examples across countries of the interplay between management and medicine. This allows us to address the aim of the study and its underlying assumptions, as well as to consider the evidence on CDs in the overall context of NPM-driven reforms in Portugal. The methods section is followed by the empirical analysis of the functioning and structure of the CDs selected for the study and the role of the hybrid manager on practising clinicians’ autonomy and exposure to hybridization. The evidence is then discussed in the light of the research questions. The concluding discussion includes reflection on the implications for practise and directions for future research.

### Patterns of hybridization in hospital organizations

Hospitals are co-habited by two structures of authority once assumed difficult to reconcile [16]. One is the rational-legal structure linked to administrative processes and rationale while the other is the charismatic structure linked to professional processes and rationale. These structures make hospitals one of the finest examples of a professional bureaucracy. Since professionals have great expertise and high status, they have autonomy to make their own decisions, thus affecting the outcomes of the organization. Professionalism is the prime coordinating mechanism of the organizational structure. Coordination mechanisms are based on the standardization of skills due to little administrative control over work and low activity planning. The role of professionalism also means formal delegation of skills to the lower levels of the organization, as well as informal delegation of skills within the same hierarchical level (e.g. patients’ referral) [17, 18].

However, professional authority goes beyond its charismatic scope given that health professionals, mostly doctors, have historically been in management positions [19]. One of the effects of the reform started in the 80s, also known as New Public Management (NPM), was to change this overly profession-based governance structure of hospitals as a result of mistrust in self-regulated knowledge and of the need to enhance the efficient use of resources [20, 21]. NPM-driven reforms have labelled a wide range of policy change worldwide in order to strengthen administrative authority in public services [22–24]. Particularly in the hospital sector this has been accomplished by limiting professionals’ room for manoeuvre through managerial tools and guidelines or their authority through the split between clinical and management duties [25]. Unsurprisingly, the number of general managers rose considerably across all management levels of hospital organizations during this time [26].

Yet, health professionals continued to resist NPM-driven reforms [27], and no clear benefits of the split between clinical and management duties were found as to the quality of care and effectiveness of decision-making [28]. Despite differences across countries in timing, pace and goals, these outcomes have been generally described as a turning point in policy agenda in which mostly medicine but also nursing were again co-opted into management positions [29]. The literature refers to this as hybrid leadership or hybrid management. The term “hybrid” is used to refer to managers with a professional background that are able to embody, translate, and mediate the logics of both management and professions [30, 31]. At least two main differences distinguish the current standpoint of hybrid roles from the past. One is the fact that hybrids are expected to have skills in management, and the chain of command is expected to result in actual changes in work models and professionals’ accountability. The other difference is the blur of professional and managerial values. Freidson [19] originally defined professionalism as a normative conception of specialized knowledge that should be self-organized and controlled apart from management and markets in order to provide society with especially important services like those linked to health care.

Resulting from hybridization, the concept of professionalism has evolved to better articulate professional and organizational logics as illustrated in organized professionalism [32]. Although it is no longer possible to reflect on professions and organizations as detached from one another, it remains controversial, particularly for clinicians in hospital organizations, their link to organizational dynamics and processes.

### Changing contexts for hybridization: the example of clinical directorates

Although different types of hybrids have been experienced in different organizational structures, CDs have been found to be in several countries the finest examples of doctors taking managerial positions in hospital organizations [26].

CDs are middle-line organizational structures that consist of autonomous multidisciplinary frameworks of care delivery, where clinicians assume management roles [33]. The existing evidence on CDs generally highlights that, on the one hand, hybrid managers experience ethical dilemmas in combining clinical and management decisions [34] and, on the other, clinicians are reluctant to embrace management duties due to the loss of autonomy in decision-making [26] or lack of teamwork effectiveness [35]. However, little attention has been devoted to the role of hybrid managers as key in determining organizational change itself, namely the functioning of CDs. The literature often opts for institutional readings focused on broad influences linked to politics, economy, science, or technology [36], while professionals are relegated to a marginal position as to how they relate to and make use of such influences at the workplace level.

Perhaps more important is to find that CDs have been roughly defined as “a move from professional bureaucracy to a divisionalized form” [33: 10]. This definition is far from being clear in the light of the non-articulation between divisionalized structures and professional bureaucracies as described in the literature on organizational configuration [17]. In sum, little is known about the effects of CDs on the configuration of hospital organizations as a professional bureaucracy model.

### The aim of the study and underlying assumptions

This article proposes to go deeper into the daily reality of CDs while illustrating an organizational structure where management and medicine are deeply embedded in each other. It seeks to witness the process of institutional change underpinning the creation of such an organizational configuration by highlighting the impact of hybrid managers on the organization’s functioning and on practising clinicians in terms of their autonomy in decision-making and exposure to hybridization. The aim is to fill the gap in the current knowledge on hybridization and the functioning of CDs as organizational structures where medicine and management stand particularly close together. As to hybridization, it has been simply assumed that clinicians align their professionalism with managerialism (the normative value system of management); and that managerialism is the primary driver of change in view of organizations’ growing commitment to cost-containment, administrative predictability, and awareness of users’ needs and rights [37]. As to the functioning of CDs, it is still unclear the connection between professionalism and divisionalized forms given that the literature considers these organizational dynamics to be incompatible.

Our contribution to the debate is structured around the following research questions. First, it is intended to examine how the interplay between professional and managerial logics embodied in hybrid managers shape the functioning of CDs concerning their structure and processes. As mentioned, although CDs have been long established in several countries, few attempts have been made to incorporate them into the literature on organizational configurations. Secondly, it is intended to show the effects of hybrid managers on practising clinicians’ autonomy and relationship with management. In other words, it seeks to find whether hybrids contribute to the hybridization of clinicians with no managerial duties or whether they act as gatekeepers of traditional forms of professionalism, thus keeping management outside of professional boundaries.

These research questions build on the following theoretical arguments. One argument is that organizations are considered as sites of contingent dynamics in which macro-level policies are subject to open-end processes of negotiation and conflict among and within professions. Not surprisingly, alliances and disputes between professionals and managers [38] are expected to take place at the workplace level, and the managerial roles taken by professionals are re-stratifying professions in complex ways [39, 40].

Another argument is that clinical and managerial values are not necessarily in opposition to each other, given that professions and organizations are similarly exposed to the pressure for increased performance [37]. Recent evidence has, however, challenged the assumption of linear causality between the instruments of governance and the effects on the relationship between medicine and management [41]. Additionally, few attempts have been made to include national-based policies under which clinicians get involved in management [26]. NPM-driven reforms more generally and CDs more specifically have been reported across the world around basic principles of private sector-style management, measurement, top-down targets, ‘quasi-markets’, and quality improvement initiatives. Yet, national variations should not be underestimated. In some countries, clinicians have been able to protect self-regulated spaces inside organizations despite the growing exposure to mechanisms of organizational control and accountability to other players, including allied health professionals, general managers, and patients [42]. Possible explanations include the fact that clinicians are able to filter and make use of broad managerial interventions [3, 43] or that medical professionalism comprises different values [44]. Therefore, instead of simply assuming a convergence between professional and managerial logics, it should be tested how clinicians relate to the normative value system of management. Ultimately the goal is to contribute to the opening of the black box of the interplay between medicine and management in hospitals containing a CD structure.

### The reform pathway of hospital management in Portugal

The Portuguese healthcare system is structured around a tax-funded NHS since 1979 that combines public financing and provision of care. Historically, the governance structure of hospitals has been overly centralized in clinicians, whether or not they have formal skills in management [45]. Growing concern with the quality of care and transparency in decision-making has led to several policy changes in the general assumptions of NPM. The first reforms date from the late 90s and then became systematic. They were aimed at ensuring hospitals greater autonomy from national and regional levels and a split between clinical and management roles [46]. After 2002 most Portuguese public hospitals have undergone similar institutional changes, which are summarized in Table 1. In one word: the broad political standpoint is similar to that in the UK during the Griffiths’ report in the early 80s, which translates into reforms aimed to increase the opening of management positions throughout the hierarchy line of hospitals and the admission of general managers to those positions.

**Table 1:** Summary of policy change in Portuguese hospitals

Policy change	Examples
Decentralization of competencies	• Purchaser/provider split: public organizations turned into public corporations and afterwards into public enterprises • Flexible models of human resources management (individual labour contracts; appraisal system; culture of meritocracy) • Accounts controlled by a single supervisor (board of directors) appointed by the Ministry of Health • Directors of departments appointed among the most qualified doctors by the hospital’s board of directors
Financing control	• Performance-based funding (DRGs) • Non-compliance with contracted activity penalizes the following year’s budget • Budget control tools at the organizational and ward levels • Evaluation in accordance with quantitative criteria
Managerial control	• Evidence-based procedures (cost/benefit ratio) • Clinical guidelines (for monitoring and assessing incentives and sanctions) • Administrative approval of medical decisions • Opening of management positions in departments (middle-level structures of control) • Hiring of general managers
Deregulation of the labour market	• Rise in individual contracts, short-term contracts, and service provision • Freezing of professional careers • Increase in wage variation within health professions
Quality and safety controls	• Monitoring technological tools • Quality reports • Internal and external audits and benchmarking • Procedures to monitor malpractice

CDs have emerged in this context but reflect the overall misunderstanding in terms of policy agenda regarding the desired position for clinicians, allied health professionals, general managers, and patients in the governance structure of hospitals. On the one hand, accountability and transparency have been pursued with a view at diluting medical authority. On the other hand, decision-making has not become more inclusive of allied health professionals and patients [47], and there are no specific criteria for those who are expected to play as hospital managers, including the obligation to hold degrees in management. As a result, no data is available to infer the profile of hospital managers.

CDs have been reported to exist in hospitals since the 90s [48]. Their establishment is regulated by the Ministry of Health under the expression of interest by the hospital’s board of directors in articulation with the senior consultant who is later appointed as the CD’s clinical director. The clinical director enjoys full autonomy and control over both clinical and management duties and may be assisted by an administrator on matters related to accounting, interface with the hospital’s board of directors, and so on. The Portuguese legislation refers to CDs as the most innovative business-like experience in public hospitals that helps achieve greater efficiency of resources while giving users improved access to cutting-edge healthcare services and ensuring professionals’ commitment and accountability to the organization [48]. A specific funding scheme different from the hospital includes overpayment of procedures and increased salaries based on target-meeting and contracting, thus making CDs the most attractive organizational model to clinicians.

The evidence is not clear regarding the extent to which the business-based governance model of CDs has shaped clinical values and autonomy. At the macro level, the engagement of medical associations and unions with management is ambivalent and no significant changes have been made in medical education. At the micro level, policy implementation has been fragmented and the experiences are hazy.

CDs in the Portuguese healthcare system are worth exploring against other known cases in Europe with respect to hybrid roles and hybridization [26]. On the one hand, the pathway of reforms in the governance of hospitals in Portugal did not emerge from a broad consensus nor did it involve effective roles by allied health professionals as in countries like Denmark. On the other hand, differently from countries like England where few management positions were filled by doctors and the admission of general managers increased clinical and financial accountability, in Portugal clinicians have consistently been interested in filling management positions. Evidence further suggests that they have been given autonomy in terms of both clinical and financial tools of control [45].

## Methods

The study was designed to witness the process of institutional change from the inside and while that process was underway. Accordingly, evidence is not intended for comparison or extrapolation. Rather, it seeks to provide in-depth knowledge on day-to-day interactions that neither cross-country comparisons nor quantitative data are able to provide. A multi-step criterion for the selection of the hospital(s) was followed. First, it should be publicly funded so that the interplay between State/managerial regulation and professional medical self-regulation is more intense. Second, it had to be going through institutional change at the time of data collection so that it could be possible to test the interplay between the two regulation logics. Third, it had to comprise a CD structure in which a hybrid manager is responsible for both clinical and managerial decisions.

### Data collection and analysis

The research was carried out in the only public hospital undergoing change at the time of data collection into a public enterprise, and it was limited to the surgery department where the establishment of a CD was being negotiated. Data collection comprised semi-structured interviews complemented with observations. By combining these techniques the purpose was to relate discourses and behaviours previous to and during the establishment of the CD, in addition to ensuring the accuracy of the information collected during the interviews. Interviews comprised a list of bullet points addressing the same analytical dimensions as for the observations (Table 2).

**Table 2:** Summary of the process of data collection

Analytical dimensions	Place of observation	Interviewees
Interplay between the hospital’s board of directors and the CD’s clinical director	Formal and informal meetings (hospital’s board of directors and CD)	General managers Clinical director
General managers’ interference in surgeons’ work	In-room consultations Operating rooms Informal talks in random places	General managers Practising surgeons
Clinical director’s interference in surgeons’ work	In-room consultations Operating rooms Informal talks in random places	Clinical director Practising surgeons
Surgeons’ view of their practise and autonomy	In-room consultations Operating rooms Informal talks in random places	Practising surgeons
Change in the delivery of care (teamwork, communication flows, selection of cases, decision-making on clinical cases)	Clinical cases meetings In-room consultations Operating rooms	General managers Clinical director Practising surgeons
Which managerial and clinical tools are used and who controls them	Formal and informal meetings (hospital’s board of directors and CD) Clinical cases meetings	General managers Clinical director

Direct observations were made on a regular basis, at least 20 h per week (nearly one year). Professionals were informed about the aim and scope of the research and their anonymity was guaranteed. The research team had access to daily activities, including those taking place in restricted areas. Field observations were recorded manually in a field diary while conducting the observations. The field diary required two writing phases: in the first, the text was written up in full so that unexpected connections between events that might contradict the existing literature could be captured. In the second phase, the writing of observations followed the analytical dimensions relevant to the study.

As to the interviews, 18 in-depth, semi-structured interviews were made with all general managers on the hospital’s board of directors (*n* = 3) and surgeons (one to the clinical director plus 14 to practising surgeons including residents, clinical assistants, associate specialists, and senior consultants).

Allied health professionals were excluded from the study, because during the observations it became clear that surgeons were the only professionals negotiating the establishment of the CD with both the hospital’s board of directors and the Ministry of Health. Moreover, the study focuses specifically on its effects on medical professionalism.

The interviews took place at the respondents’ workplace and lasted between 60 and 120 min. Interviewees authorized the use of a recording device and were informed that the contents would be transcribed in full.

The field diary and interviews were later transcribed in full and processed with the MAXQDA software for qualitative content analysis, which was used to compile information both by dimension and by respondent.

## Results

### Organizational features of the CD: its funding and functioning

A CD is a medical department with a clinical director to which management duties are formally delegated by the hospital’s board of directors:

It represents the financial and administrative autonomy that would be ideal for me. It would allow me to hire according to my specific targets, I could invoice our work and then pay our salaries. Part of the profits we would bring to the hospital would ultimately be distributed for investment and staff. […] This money would enable me to hire my own specialists and also staff to complement my multidisciplinary group. […] (Clinical director)

Although its establishment is formally conducted by the hospital’s board of directors together with the Minister of Health, the senior consultant, who is appointed as the CD’s clinical director, plays a key role in this process:

[on the establishment process of the CD]

Everything depends on me. I’ve set the conditions and timing with the former minister [of health] and the current [minister] will definitely comply with the arrangement […] the hospital’s board of directors has no word in this. I’m in control of everything. (Clinical director)

Practising surgeons support this organizational change in the expectation of profiting from their practise in view of the overpayment procedures CDs are subject to. The procedures performed here are among the most costly to the NHS:

What keeps me working here? I can actually make money from my activity as a public servant (surgeon #3)

Medical staff and allied health professionals are in exclusive dedication to the CD. The same happens to administrative staff including general managers whose role is to support clinical decision on issues such as hiring staff, defining performance targets, and deciding the investment policy in medical technologies. As a result, the CD requires the creation of an autonomous organizational structure inside the hospital comprising a strategic apex, a specific middle line, support staff, and an operational core with which the hospital contracts the activity (performance, costs, quality indicators, and so on).

The more the time I spend here the more I realize I am in an organization inside another organization. The administrative procedures here are not common to other departments, nor are the targets, mechanisms of accountability or human resources management. (note of the field diary)

The clinical director is expected to perform a business-like management that combines clinical and financial control and the daily activity of staff is expected to be optimized according to the productivity targets set by the clinical director. The underlying assumption is that the more activity is performed and the higher the quality of the procedures, the more the CD profits and reinforces its scientific prestige.

During the observation stage it became evident, however, that the clinical director has not strengthened tools of control, namely those related to management (note of the field diary). Obviously, the clinical director seeks to ensure predictability in surgeons’ performance regarding quality and patients’ safety. The issue is that practising surgeons reveal a strong commitment to those targets even in the absence of formal mechanisms of imposed control. This translates into an informal work model based on the clinical director’s selection of the staff. Some of the surgeons working here are former trainees of the clinical director while the others have been hired directly by him. In sum, the absence of formal mechanisms of control is replaced with close ties of commitment, trust, and shared goals of quality and profit between the clinical director and all staff working in the CD.

General managers on the hospital’s board of directors are supportive of the creation of the CD even at the expense of losing administrative control. Such interest relies on the CD’s financial and production targets that are also key to the hospital as a whole. This argument is better understood against the NHS internal market: public providers have to compete to attract funding and patients, and cuts in public funding and benchmarks are based on quantitative criteria. Subsequently, informal alliances and disputes between general managers and clinics become visible:

The more they [surgeons] perform the more money they bring to the hospital. We simply need them […] (General Manager #3)

[…] we have areas of great technical expertise while others are less so: surgery is strong and powerful, and it enjoys national and international prestige […]. We gave them more beds and improved comfort […] in detriment to other departments. […] So the decisions weren’t taken so as to give the same to all. No!!! (General Manager #1)

On the other hand, the CD involves greater differentiation and conflicts within the medical profession, in addition to highlighting the role of the clinical director in determining the organizational arrangements of the hospital as a whole:

Doctors of other departments frequently oppose the CD… they contest different specific rules and funding opportunities but mainly the fact that not all medical specialties can perform as quickly and as predictably so that they could also apply for CDs in their departments. (note of the field diary)

The hospital has departments that make no sense. I need others that can help the CD and not the ones that take the hospital 100 years back. […] The board of directors needs to understand […] there are departments that have not adapted their methods to our needs [of the CD]. […]. The key is to know how I can convince the [hospital’s] board to make the right changes in the hospital […] it’s something I’ve been working on… (clinical director)

Broadly speaking, the governance structure and functioning of the CD rely on features of medical work. Only those medical areas involving high technological differentiation and relatively routinized procedures that allow high patient turnover meet the principles of standardization required to the performance-based funding scheme of CDs. By this mean internal re-stratification in medicine is intensified.

### The influence of the hybrid manager on practising surgeons’ performance

As mentioned above, the clinical director’s control over staff is informally exerted. This evidence is now reinforced through practising surgeons’ response to perceived changes in their work, if they felt more accountable and to whom. Surgeons were unable to mention changes to their work, in addition to spending more time in the hospital. Furthermore, they all said that they feel more accountable to the clinical director but mostly concerning patient safety. By no means this implies that surgeons feel more controlled by the clinical director in their decision-making. For instance, a large number of clinical meetings or informal talks take place in the absence of the clinical director to discuss procedures and set joint decisions concerning cases of high complexity (note of the field diary). The clinical director focuses more on meeting overall goals than controlling the processes in detail, and he trusts the decisions made by the team of surgeons. It is worth to emphasize that practising surgeons were either trained or hired by the clinical director. In relation to financial control no additional tools were found or mentioned. The fact that surgeons’ payment is performance-based automatically makes them accountable to improve individual targets even in the absence of rules imposing such criteria. When asked about the possibility of introducing additional financial tools, surgeons generally do not realize how it can improve the current standards of quantity and quality.

Common to all surgeons is the fact that tools of control are mentioned only in relation to the clinical director, since they are not fully aware of the role or even presence of the hospital’s board of directors in their daily life. This results in the fact that surgeons’ projects and interests lie exclusively in their commitment to the CD rather than to the hospital as a whole:

The hospital has never given me anything. [The managers at the board of directors] weren’t responsible for my training. It’s the clinical director that I’m indebted to… I owe him a great deal; but no, I don’t owe the hospital anything! (Surgeon #6)

[…] the hospital is less important [than the CD]. I’ll go wherever it is established. (Surgeon, #1)

Both excerpts illustrate effects of divisionalization as described in the literature but clearly in articulation with professional values. First, because surgeons show strong bonds with their peers. Second, because they seem to widen the gap with the managers at the hospital’s board of directors. Third, because their commitment is to the CD rather than to the hospital as a whole, which is particularly enlightening if one considers the public nature of this hospital. Surgeons’ performance is thus structured more according to specific professional criteria and less to overall goals related to the mission and vocation of the NHS.

## Discussion

### The clinical directorate within the hospital organization: arguments of the divisionalized professional bureaucracy model

Despite the long-term tradition of CDs in different countries, few attempts have been made to incorporate these divisionalized structures into the overall debate on organizational configuration, particularly their link to the core features of the professional bureaucracy model. The issue is that this combination is considered to be incompatible: “What makes a structure divisionalized is managerial or unit autonomy, not professional autonomy” [17: 215]. Also recent analyses do not elucidate the extent to which the CD implies articulation, or instead replacement, between the features of professional bureaucracies and divisionalized forms [33].

Briefly, the non-articulation between both structures is explained by the pressures underpinning them (professionalism and balkanization) which are summarized as follows (see left and right columns of Table 3): decision flows (bottom-up vis-à-vis top-down); planning and control systems (little vis-à-vis extensive); support staff (intended to support professionals vis-à-vis the existing split between headquarters and divisions with a strong emphasis on managerialism); autonomy (professional vis-à-vis managerial). In addition, there is no possible link particularly in the public sector since the divisionalized form is defined as the evolution of large mechanical bureaucracies towards adjustments in the market of the lucrative private sector.

**Table 3:** Analytical features of professional bureaucracy/divisionalized form and empirical evidence of the divisionalized professional bureaucracy

	Professional bureaucracy	CD as a divisionalized professional bureaucracy	Divisionalized form
Key coordinating mechanism	Standardization of skills	Standardization of skills and outputs	Standardization of outputs
Key part of organization	Operating core	Middle line controlled by the operating core	Middle line
Support staff	Elaborated to support professionals	Split between headquarters and divisions to support professionals	Split between headquarters and divisions
Pressure	Professionalism	Balkanized professionalism	Balkanization
Formalization of behaviour	Little formalization	High formalization	High formalization
Planning and control systems	Little planning and control	High performance control	High performance control
Flows of decision making	Bottom-up	Top-down (with autonomy)	Top-down (with autonomy)

Adapted from Mintzberg [17, 18]

The evidence suggests articulation, instead of replacement, between the features of professional bureaucracies and divisionalized forms. Accordingly, the CD under study seems to exemplify a divisionalized professional bureaucracy structure not yet documented in the literature (as detailed in the centre column of Table 3). First, it results from the vertical decentralization of duties to the hybrid manager, notably the management of facilities, funding and human resources, according to a pay-per-performance scheme. Second, top-down decision flows aim to standardize outputs at the same time bottom-up autonomy is reinforced, since the hybrid manager is given autonomy to define clinical criteria and compliance with performance. Third, since it is a hybrid manager who runs the divisionalized form, the middle line management of the hospital remains under the control of the operating core. Fourth, as all staff belongs to the CD, clinical and managerial duties and guidelines are separated from hospital’s board of directors. The feature that ultimately makes the CD a divisionalized professional bureaucracy structure is the way coordinating mechanisms of balkanization and professionalism stand together. The criteria underpinning funding and quality is medical-based, in which management criteria only serve to support medical governance. However, because not all medical specialties can apply for and benefit from the conditions of CDs, re-stratification is expected to enlarge in the medical profession [49], this way creating effects of balkanization in medical professionalism (see the reference to the role of the hybrid manager in negotiating directly with the Minister of Health the establishment of the CD).

Linked to the role of the hybrid manager as key in organizational change, this discussion resonates with recent insights [41, 50, 51], according to which workplace interactions need to be emphasized in the understanding of the processes and outcomes of organizational change. In this case, the clinical director not only led to the establishment of the CD but also moulded the practising surgeons’ commitment to their work, consequently affecting their relationship with general managers and other clinicians.

### Effects of hybrid managers on medical professionalism: features of balkanized-organized professionalism

Building on CDs as an empirical illustration of a divisionalized professional bureaucracy structure, the interaction between medical professionalism and managerialism requires a different analytical positioning. The clinical director defines, monitors and assesses both clinical and financial tools of control through an informal management style based on trust and share of goals with practising surgeons. Hence it can be assumed that the clinical director acts as a gatekeeper of external influences, namely of the hospital’s board of directors as to how practising surgeons perform daily.

In this sense, the dependency pattern of professionalism on organizational spaces and dynamics is confirmed [32, 37]. However, unlike often assumed in the literature on hybridization, any conclusion concerning possible effects of managerialism on professionalism is not deducted. To put it differently, empirical evidence confirms that organizations serve as sites for professional development; that the nature and scope of professional work is somehow dependent on organizational processes; and that professionals take new organizational roles aligned with managerial values. However, these three scopes linking professionalism to organization-based dynamics do not confirm linear alignment of professionalism with managerialism. Surgeons’ professional values and interests remain autonomous from those of management given that no differences were introduced in the CD related to work models, growing administrative control or loss of autonomy in decision-making. The hybrid manager is key to understand the limited exposure of practising professionals to hybridization. The hybrid manager did not strengthen financial tools of control, and clinical tools remained discretionary-based and independent from the hospital’s board of directors’ intervention. As suggested in other studies medical authority can get reinforced in organizational settings subject to NPM-driven reforms [27, 38, 42, 45, 49].

Furthermore, surgeons’ enhanced performance does not in their clinicians’ minds translate into hybridization. Growing productivity is correlated with expertise and skills acquisition as well as with the growing capacity to perform autonomously from the clinical director’s control. They realize that the more they perform the better they do it; and that their purpose is to meet the clinical director’s standards and expectations.

On the other hand, the underlying influence of the nature of medical procedures in all these dynamics should not be disregarded. As seen, not all medical specialties can perform as quickly and as predictably as surgery. In this sense, organized professionalism needs to be reassessed along with the so-called *balkanized* effects. The shift lies in considering the organizational scope of professionalism not in relation to the organization but in relation to one of its divisionalized parts. Balkanized-organized professionalism is not opposed to, and builds on the original definition of, organized professionalism. The difference is that professionalism is aligned with but not limited to the organization as it is often assumed in the literature. Balkanization is introduced in two complementary ways. One is to consider balkanized professional interests, which result from processes of gatekeeping as those enacted by the hybrid manager. Consequently practising professionals pursue interests according to specific motivations set by the professional leader, regardless of the organization’s overall administrative functioning. The other complementary way is to consider balkanized structures in the organization. As mentioned before, the goals and processes undertaken in autonomous units within the organization may diverge from each other and from the organization’s mission as a whole. The mission and vocation of the CD do not necessarily converge with those of the hospital. In sum, what makes organized professionalism balkanized is the fact that it is not common to either professions or organizations.

## Conclusion

Building on the experience of this CD, which differs from that reported in Anglo-Saxon and Scandinavian countries [26, 33], the discussion on hybridized managerial roles highlights the need to consider more seriously the impact of specific features of the governance models of health systems. However, few attempts to clarify such empirical variation have been made to include national-based policies in the creation of hybrid organizational configurations. For this reason, the key requirement in this study was to capture organizational change from the inside and while the process was underway by combining qualitative research tools that neither cross-country comparisons nor quantitative data have been able to provide.

Unlike often suggested, the establishment of this CD reveals the extent to which organizational structures comprising hybrid roles can be driven and supported by clinicians. Although CDs are expected to lead to greater accountability over professionals in general and to dilute medical authority in particular, data has shown that policy orientation is subject to specific processes controlled by clinicians at the workplace level. Regarding the relationship between medicine and management, policy setting and expected outcomes are not correlated as one might assume. Possible explanations include unchanged governance mechanisms at the macro-level, thus exposing organizational change to micro-level variations; lack of involvement of regulatory professional boards in policy setting; few changes in medical training in terms of management skills; or the charismatic authority clinicians still enjoy among the population in general.

Contingency in the establishment of this CD also highlighted the key role of the hybrid manager in determining the extent to which practising professionals are more accountable to managerial criteria and consequently the ensuing effects on their autonomy. The overall conclusion is that although medical and managerial values link to each other, clinicians are able to make use of and reconfigure managerial criteria according to specific interests. Ultimately, medical autonomy and authority may be reinforced in organizational settings subject to NPM-driven reforms. Yet, such ability is rather specific to medical specialties thus contributing to a growing re-stratification in medicine. The discussion suggests forms of balkanized (divisionalized) medical professionalism reinforced by hybrid roles.

Several implications for practise can be drawn, thus highlighting directions for future research. First, it has been confirmed that hybrid managers enhance clinical and management outcomes, and that they can perform a key role in determining organizational change. Second, clinicians’ commitment to work is not correlated with growing managerial intervention in daily processes; their performance is rather based on financial incentives. In the absence of managerial rules, professional leaders play a key role in clinical safety and quality, particularly in the selection and management of human resources. Third, unintended consequences of divisionalized structures may arise regarding the functioning of public hospitals, given that professionals are less committed to the overall mission and vocation of the hospital as a whole. Such consequences may include lack of articulation in care, growing differentiation among public servants, and greater conflicts among wards for improved conditions.

## Abbreviations

CD: clinical directorate
NPM: New Public Management
